# Sepsis in Africa: practical steps to stem the tide

**DOI:** 10.11604/pamj.2015.21.323.6462

**Published:** 2015-08-31

**Authors:** Akaninyene Otu, James Elston, Emmanuel Nsutebu

**Affiliations:** 1 Department of Internal Medicine, University of Calabar,Nigeria; 2 Public Health, Yorkshire Deanery, Leeds, United Kingdom; 3 Tropical and Infectious Diseases Unit, Royal Liverpool University Hospital Trust, Liverpool, United Kingdom

**Keywords:** sepsis, Africa, guidelines

## Introduction

Sepsis is a leading cause of morbidity and mortality worldwide and particularly in Africa where awareness is low and resources are limited. There are limited reports on the epidemiology, management and outcomes of the sepsis syndromes from Africa. However, this region is likely to account for a significant proportion of the global burden of sepsis which goes unrecognized. It is imperative to address this through research, increased awareness, capacity building and introduction of practical clinical guidelines. Infections are responsible for an estimated 300 million annual deaths worldwide, the majority from developing countries [1]. Sepsis can be triggered by almost any infection and is responsible for an estimated 8 million annual deaths worldwide [1]. In the United Kingdom (UK), sepsis is the third most important cause of death in hospital with an average management cost of £20,000 per admission [2]. Given the high incidence of Human Immunodeficiency Virus (HIV) and other infections in the African continent, it is likely that the burden of sepsis is at least equal if not higher than estimates from Europe and North America.

## Why are sepsis related cases and deaths under-reported?

Firstly, health care professionals often miss the diagnosis or fail to document it in the clinical notes. Secondly, the World Health Organisation (WHO) Global Burden of Disease Report (GBDR) does not include sepsis as a cause of death. The GBDR is one of the leading information sources for healthcare decision-making worldwide. Although deaths from infections occur most commonly as a result of sepsis, the GBDR lists only the underlying infections as cause of death. It is therefore not surprising that sepsis occurs only as “neonatal” sepsis and is ranked 16th place, despite about 60% of deaths in children under five being due to severe infections. Thirdly, guidelines for coding of sepsis are often difficult to use especially in under resourced and busy developing world healthcare settings.

**Interventions to improve the management of sepsis**: the last decade has seen massive campaigns to improve sepsis management via targeted initiatives predominantly in developed countries. The international Surviving Sepsis Campaign (iSSC) published international guidelines for management of severe sepsis in 2004 and this was subsequently updated and condensed into two care bundles. Following the ABC of resuscitation, the following measures are recommended to be completed within three hours of presentation: measurement of serum lactate levels; obtaining blood cultures prior to the administration of antibiotics; administration of broad-spectrum antibiotics; administration of 30 ml/kg crystalloid for hypotension or serum lactate >4 mmol/l (36 mg/dl).

The iSSC guideline also has a 6 hour bundle for severe sepsis and septic shock. It consists of invasive monitoring of physiologic parameters such as central venous pressure and use of vasopressors to maintain mean arterial pressure usually in an intensive care setting. The use of such an intensive resuscitation bundle in the form of early goal directed therapy with resuscitation targeting specific physiologic goals has been shown to significantly reduce mortality in severe sepsis and septic shock when compared to standard therapy. However, recent self-reported survey data strongly suggest that these critical care measures, though beneficial, cannot be implemented in most parts of Africa due to a shortage of resources such as skilled manpower, complex equipment and drugs [3]. A recent trial in the United States of America (USA) also suggests that early recognition and resuscitation of patients with sepsis is way more important than early goal directed therapy [4]. It is therefore reasonable to focus attention on improving awareness among clinicians and developing resuscitation guidelines which are feasible within the African context. These can then be applied to patients in the early stages of sepsis to halt the progression to severe sepsis and septic shock that portend poorer prognosis. Interestingly, the basic resuscitative measures for managing sepsis are reflected in the Integrated Management of Adult and Adolescent Illnesses (AMAI) document of the WHO. This set of guidelines is aimed at first-level facility health workers and lay providers in low resource settings. Though it does not explicitly refer to sepsis, the guidelines promote the prompt intravenous (IV) fluid resuscitation, antibiotic use and timely referral in settings which are arguably akin to septic shock.

The diagnosis of sepsis can be facilitated by the use of a clinical early warning score (EWS) to aid the identification of the systemic inflammatory response syndrome (SIRS). The EWS is based on the principle that physiological deterioration precedes critical illness. It assesses physiological parameters such as systolic blood pressure, pulse rate, respiratory rate, temperature and oxygen saturations. A score is allocated to each parameter as they are measured and a score is then aggregated. The total score determines the action to be taken by the assessor thus enhancing early detection of the deteriorating patient, timely clinical response and reduction in referral delays. However attempts at validating EWSs in resource limited settings have produced conflicting results.

**Applicability of the ISSC guidelines to the African context**: this section discusses the applicability of the 3 hour iSSC bundle to the African healthcare system in greater detail.

**Lactate measurement**: serum lactate is a marker for tissue hypoperfusion and rises in patients with severe sepsis. It is associated with disease severity and mortality. In patient with sepsis, lactate is an important component of the initial evaluation and can also be used to assess response to treatment. Point-of-care testing (POCT) of lactate is now increasingly available for use in the emergency department (ED) in resource poor settings and is low-cost. The handheld POCT device which measures fingertip and whole blood lactate has been shown to be accurate and time saving when compared with reference laboratory testing in critically ill ED patients in the USA [5]. Research is needed to assess the utility of POCT lactate measurement in adult sepsis patients in a developing country setting.

**Blood cultures**: data from the iSSC suggests that performing blood cultures is independently associated with improved survival. An efficient blood culture system requires competent laboratory scientists and facilities for standard aerobic and anaerobic cultures. Availability, accessibility and affordability of blood cultures are a problem in many African countries. However, molecular techniques are in development and may improve ability to detect patients with bacteremia and sensitivity patterns in resource limited settings.

**Antibiotic administration**: prompt administration of antibiotics in severe sepsis and septic shock has been shown to reduce mortality. It is advocated that empiric antibiotics be given after blood cultures have been taken. Antibiotics can subsequently be rationalized based on blood culture results. A study from Uganda showed that empiric antibacterial therapy in sepsis was rarely concordant with blood culture sensitivities [6]. Thus, empiric antibiotic regimens need to be updated regularly based on local antimicrobial resistant trends. There is a need to develop evidence-based formularies for empiric antibiotics based on local antimicrobial sensitivity data. This could be achieved through routine collection of data or annual surveillance surveys.

## IV fluids

The aggressive use of IV fluids in sepsis can decrease duration of hypoperfusion of vital organs ultimately resulting in less end-organ damage. However despite numerous studies comparing colloids and crystalloids, research is needed to determine the ideal resuscitation fluid. Recent research suggests lower hospital mortality in patients who receive larger volumes of IV fluids within 3 hours of onset of sepsis [7]. Interestingly, both groups - survivors and patients - received the same total amount of fluid in the first 6 hours. Therefore, timely fluid resuscitation within 3 hours of onset may increase survival. However, a recent report from sub-Saharan Africa calls for caution in applying this to children. This Fluid Expansion as Supportive Therapy (FEAST) trial showed a significant increase in the mortality within 48 hours in febrile children who received boluses of albumin or saline. However critical care facilities were either limited or absent [8].

## Recommendations

To address the challenge of sepsis in Africa, the following steps should be prioritized: awareness creation and capacity building among health workers about sepsis and its management. This should promote early sepsis recognition as the features of sepsis may be subtle; an early recognition and management bundle for sepsis should be developed to reflect primary, secondary and tertiary level of care and should include the following: use of validated EWS and SIRS criteria to identify patients with sepsis; rapid administration of oxygen, intravenous fluids, antibiotics and measurement of fluid balance; lactate measurement and blood culture collection where possible.

- This bundle for sepsis management should be concatenated in user friendly guidelines that reflect actions which are feasible within the various levels of healthcare. These guidelines can then be widely disseminated with guidance notes and regular training to re-enforce the key messages and promote compliance.
- Quality assurance teams should be set up within healthcare facilities to improve record keeping and clinical coding.

## Conclusion

There is an urgent requirement to explicitly recognize sepsis as a significant cause of morbidity and mortality in Africa and make greater efforts to more precisely describe the burden of disease from sepsis. Sepsis quality improvement programmes are desperately required in Africa to provide context-specific solutions to this catastrophe.

## References

[1] World sepsis day newsletter Sepsis: prevent it, spot it, treat it, beat it.

[2] NHS England Factsheet: Implementation of the? Sepsis Six? care bundle.

[3] Baelani I, Jochberger S, Laim T, Otieno D, Kabutu J, Wilson I (2011). Availability of critical care resources to treat patients with severe sepsis or septic shock in Africa: a self-reported, continent-wide survey of anaesthesia providers. Crit Care..

[4] The ProCESS Investigators (2014). A randomised trial of protocol-based care for early septic shock. N Engl J Med..

[5] Gaieski DF, Drumheller BC, Goyal M, Fuchs BD, Shofer FS, Zogby K (2013). Accuracy of Handheld Point-of-Care Fingertip Lactate Measurement in the Emergency Department. Western Journal of Emergency Medicine..

[6] Jacob ST, Moore CC, Banura P, Pinkerton R, Meya D (2009). Severe Sepsis in Two Ugandan Hospitals: a Prospective Observational Study of Management and Outcomes in a Predominantly HIV-1 Infected Population. PLoS ONE..

[7] Melville NA Early Fluid Resuscitation Reduces Sepsis Mortality. Medscape.

[8] Maitland K, Kiguli S, Opoka R (2011). Mortality in African children with shock. N Engl J Med..

